# Correction to: Lactate modulates microglia polarization via IGFBP6 expression and remodels tumor microenvironment in glioblastoma

**DOI:** 10.1007/s00262-022-03243-z

**Published:** 2022-07-12

**Authors:** Lucia Longhitano, Nunzio Vicario, Stefano Forte, Cesarina Giallongo, Giuseppe Broggi, Rosario Caltabiano, Giuseppe Maria Vincenzo Barbagallo, Roberto Altieri, Giuseppina Raciti, Michelino Di Rosa, Massimo Caruso, Rosalba Parenti, Arcangelo Liso, Federica Busi, Marco Lolicato, Maria Caterina Mione, Giovanni Li Volti, Daniele Tibullo

**Affiliations:** 1 Department of Biomedical and Biotechnological Sciences, University of Catania, Catania, Italy; 2 IOM Ricerca, 95029 Viagrande, CT Italy; 3 Department of Medical and Surgical Sciences and Advanced Technologies, F. Ingrassia, Anatomic Pathology, University of Catania, Catania, Italy; 4 Department of Drug Sciences, University of Catania, Catania, Italy; 5 Department of Medical and Surgical Sciences, University of Foggia, 71100 Foggia, Italy; 6 Department of Cellular, Computational and Integrative Biology (Cibio), University of Trento, 38123 Trento, Italy; 7 Department of Molecular Medicine, University of Pavia, Pavia, Italy

## Correction to: Cancer Immunology, Immunotherapy https://doi.org/10.1007/s00262-022-03215-3

The original version of this article unfortunately contained a mistake. Affiliation details for Authors’ Stefano Forte, Cesarina Giallongo, Giuseppe Broggi, Rosario Caltabiano, Giuseppe Maria Vincenzo Barbagallo, Roberto Altieri, Giuseppina Raciti and Arcangelo Liso were incorrectly given.

The correct affiliations details for Authors’ Stefano Forte, Cesarina Giallongo, Giuseppe Broggi, Rosario Caltabiano, Giuseppe Maria Vincenzo Barbagallo, Roberto Altieri, Giuseppina Raciti and Arcangelo Liso should be:

2 IOM Ricerca, 95029 Viagrande, CT, Italy

3 Department of Medical and Surgical Sciences and Advanced Technologies, F. Ingrassia, Anatomic Pathology, University of Catania, Catania, Italy

4 Department of Drug Sciences, University of Catania, Catania, Italy

5 Department of Medical and Surgical Sciences, University of Foggia, 71100 Foggia, Italy

